# The impact of sedation depth on the occurrence of delirium and prognosis in intensive care unit patients: a meta-analysis

**DOI:** 10.3389/fmed.2026.1823354

**Published:** 2026-05-04

**Authors:** Haining Zheng, Zhongpeng Yin, Qiong Zhang

**Affiliations:** 1 Department of Hyperbaric Oxygen, Jinling Hospital, Affiliated Hospital of Medical School, Nanjing University, Nanjing, China; 2 Department of Obstetrics and Gynecology, Jinling Hospital, Affiliated Hospital of Medical School, Nanjing University, Nanjing, China

**Keywords:** delirium, depth of sedation, ICU, meta-analysis, prognosis

## Abstract

**Background:**

Delirium and poor clinical prognoses are prevalent among ICU patients, particularly those requiring mechanical ventilation. While sedation is a cornerstone of intensive care, the impact of its depth remains controversial; deep sedation is often linked to adverse outcomes, whereas light sedation may improve recovery trajectories. We conducted a systematic review and meta-analysis to clarify the association between sedation depth and delirium incidence, mortality, and ICU length of stay (LOS).

**Methods:**

Following PRISMA 2020 guidelines and PROSPERO registration (CRD420251054377), we searched PubMed, Embase, Cochrane Library, and Web of Science for randomized controlled trials (RCTs) and cohort studies published through June 17, 2025. Two reviewers independently performed data extraction and quality assessment using RoB 2 for RCTs and the Newcastle-Ottawa Scale (NOS) or ROBINS-I for non-randomized studies. Evidence quality was evaluated using the GRADE framework.

**Results:**

Eleven studies (6 RCTs, 5 cohorts) involving 3,466 patients were included. Meta-analysis using a fixed-effects model demonstrated that deep sedation was significantly associated with a higher incidence of delirium (OR: 1.34; 95% CI: 1.15–1.57; *P* = 0.0001; *I*^2^ = 18%). Deep sedation was also significantly linked to increased mortality (OR: 1.71; 95% CI: 1.32–2.21; *P* < 0.0001; *I*^2^ = 1%) and prolonged ICU LOS (MD: 1.17 days; 95% CI: 0.54–1.81; *P* = 0.0003; *I*^2^ = 23%). A secondary analysis of two studies comparing dexmedetomidine to other sedatives was limited by the small number of included studies and low statistical power, such that current evidence remains insufficient to draw a definitive conclusion regarding its effect on delirium incidence (OR: 0.93; 95% CI: 0.57–1.51; *P* = 0.77). GRADE assessment rated the overall quality of evidence for primary outcomes as Moderate, downgraded primarily due to residual confounding in cohort studies.

**Conclusion:**

Deep sedation is significantly associated with an increased risk of delirium, higher mortality, and extended ICU stays. While these associations are consistent across study designs, clinicians should account for the multifactorial influences on these outcomes. Implementing sedation protocols that prioritize lighter sedation depth may be associated with improved ICU patient prognoses.

**Systematic review registration:**

https://www.crd.york.ac.uk/PROSPERO/view/CRD420251054377, identifier CRD420251054377.

## Introduction

Delirium is an acute and fluctuating change in mental state that is characterized mainly by inattention, disordered thinking, and altered levels of consciousness and is usually accompanied by cognitive impairment or perceptual abnormalities (1). It is a common complication in ICU patients. In mechanically ventilated ICU patients, the incidence of delirium is as high as 70–80%, whereas it is approximately 20–50% in nonmechanically ventilated ICU patients (2). Notably, delirium can significantly increase the mortality risk of ICU patients. Previous studies have shown that the mortality rate of ICU patients with delirium is 2–3 times higher than that of patients without delirium (3). Moreover, the length of stay of patients with delirium in the ICU and hospital is significantly prolonged (4).

Sedation refers to the use of medications to reduce patient anxiety, agitation, and pain and to promote a state of calmness and relaxation (5). In ICU patients, sedation helps relieve patient discomfort caused by various invasive procedures, mechanical ventilation, and the stressful environment of the ICU (6). Accurate assessment of the depth of sedation can ensure that patients are in an appropriate sedated state, alleviating their pain and anxiety without hindering necessary medical procedures and disease observation (7). Moreover, this plays a crucial role in preventing complications caused by inappropriate sedation, such as delirium and respiratory depression. The Richmond Agitation-Sedation Scale (RASS) is a commonly used tool. It ranges from + 4 (combative) to −5 (unarousable), with 0 representing alert and calm (8).

Regarding the relationship between sedation depth and delirium, studies have indicated a complex connection (9). Some studies have shown that there is a close connection between the depth of sedation and the occurrence of delirium (10, 11). Oversedation can increase the incidence of delirium. Inappropriately deep sedation may increase the risk of delirium (12). Prolonged and deep sedation can disrupt the normal physiological and neurological functions of the brain, leading to cognitive impairment and an increased likelihood of delirium. However, excessive sedation may not effectively relieve patients’ discomfort, which can also contribute to the development of delirium due to high levels of stress and agitation (13). However, other studies suggest that there is no connection between the depth of sedation and delirium (14, 15), demonstrating that there is no significant difference in the incidence of delirium between patients with deep sedation and those with mild sedation. Notably, existing research has primarily focused on delirium incidence while overlooking associations with mortality and ICU length of stay (LOS), and few studies have evaluated the specific role of dexmedetomidine in this context. Additionally, recent studies published after 2020 (16, 17) have provided new insights into sedation depth and delirium, which remain unintegrated in current meta-analyses. Therefore, a meta-analysis was conducted to clarify the risk of delirium occurrence under different depths of sedation. This meta-analysis aimed to systematically synthesize evidence from available randomized controlled trials and cohort studies on the association between sedation depth and delirium incidence, mortality, and ICU length of stay in ICU patients, so as to clarify the direction and magnitude of the effect of sedation depth on key clinical outcome.

## Methods

The report of this investigation strictly follows the PRISMA extension statement. All aspects of the reporting process were carried out in line with the detailed requirements set forth in its Checklist and Explanations. The methodology of this research was registered in PROSPERO (CRD420251054377).

### Search strategy

RCTs and cohort studies that evaluated the impact of sedation depth on the occurrence of delirium and were published until June 17*^th^*, 2025, were searched in databases such as PubMed, Embase, the Cochrane Library and Web of Science. The keywords used and the search strategies used are listed in Supplementary Table 1.

### Inclusion and exclusion criteria

Eligible studies for this analysis had to meet several criteria: Both RCTs and NRSs (matched-pair analyses, retrospective cohorts, pre-post quality improvement studies) were included to integrate causal evidence (RCTs) and real-world practice evidence (NRSs), with adult ICU patients (age ≥ 18 years) including those admitted due to various causes like postsurgical cases, severe trauma, septic shock, and cardiopulmonary failure. The interventions needed to involve different depths of sedation, along with clear validated assessment methods such as the Richmond Agitation-Sedation Scale (RASS) and Sedation-Agitation Scale (SAS); delirium, reported via validated tools (e.g., Confusion Assessment Method for the ICU [CAM-ICU]), had to be one of the outcomes. Studies were included if they evaluated sedation depth and clinical outcomes in high-risk patients (including critically ill ICU patients and perioperative high-risk patients, such as elderly individuals undergoing fracture repair) and reported valid data on the association between sedation depth and delirium, mortality, or length of stay. On the other hand, studies were excluded if there was no significant difference in sedation depth between patient groups, if they had incomplete data or unclear methodology, or if they were non-English articles.

### Outcomes measurement

The primary outcomes of this study included the occurrence of delirium and mortality. Meanwhile, secondary outcomes consisted of the length of stay (LOS) in the ICU.

### Data extraction

A customized data extraction form, inspired by the Cochrane Library’s data extraction template, was created. Two reviewers independently conducted the data extraction. For each trial meeting the inclusion criteria, various information was carefully obtained. This included the attributes of trial subjects such as age, sex, and the specific inclusion and exclusion criteria of the trials. Details regarding intervention types were also extracted, like sedative drugs, dosage, frequency, and RASS (or SAS) scores. Additionally, reported outcomes, the duration of follow-up, and any adverse events occurring during the trial period were gleaned.

### Definitions of key variables

Deep sedation was uniformly defined by the core clinical criterion of “inability to respond to verbal stimuli” across all studies, with operationalization matching each study’s assessment approach: RASS ≤ −3 or SAS 1–2 for scale-based evaluations; BIS≈50 (corresponding to unresponsiveness to noxious stimuli) for perioperative electroencephalographic monitoring; and clinical indicators for sedation protocol studies-all aligned with scale-based deep sedation criteria. Although these scales (RASS, SAS, BIS) and clinical descriptions are not fully equivalent metrics, all reflect a state of reduced arousal consistent with deep sedation in clinical practice. Delirium was defined via DSM criteria or ICU-specific tools (e.g., CAM-ICU), marked by acute mental status changes, inattention, and disorganized thinking/altered consciousness. Secondary outcomes included 28-day ventilator-free days, ICU/hospital length of stay, and all-cause mortality (28-day/hospital discharge).

### Quality assessment of RCTs and non-RCT studies

The assessment of the RCTs was conducted via RevMan.5.4, while the evaluation of the non-RCT studies was carried out according to the Newcastle-Ottawa Scale (NOS) (18). Two reviewers, Haining and Zhongpeng, independently carried out the assessment and validation of the quality of the included trials, strictly following the guidelines from the Cochrane Handbook for Systematic Reviews of Interventions. If there were any divergences between Haining and Zhongpeng during this process, a third reviewer, Qiong, was consulted. Qiong’s task was to deliberate on conflicting views and facilitate the achievement of a consensus, thereby guaranteeing the precision and objectivity of the quality evaluation for the trials included in the study.

Two independent reviewers (Haining and Zhongpeng) assessed the risk of bias using design-specific tools, with consensus resolution for discrepancies. For 7 included RCTs, the RoB 2 Excel macro tool (19) evaluated 5 domains (randomization, intervention deviations, missing data, outcome measurement, selective reporting), rating each as svaluated 5 domains (randomization, intervention denon-randomized studies (NRS), the ROBINS-I tool assessed 7 domains (confounding, selection bias, etc.) against the omains (confounding, selection bias, viations, missing data, outc(e.g., APACHE scores, baseline delirium). A pre-specified subgroup sensitivity analysis compared delirium effect sizes between RCTs and NRS to quantify design-related bias impact.

### Statistical analysis

The meta-analyses were performed via the Cochrane Collaboration software RevMan5.4. Binary data are presented as odds ratios (ORs) accompanied by 95% confidence intervals (CIs). Continuous data are presented as the mean difference (MD) with the standard deviation (SD).

Given that both randomized controlled trials (RCTs) and observational cohort studies reflect sedation practices in real-world intensive care units, we included both study designs to enhance generalizability. However, we prespecified subgroup analyses by study design (RCT vs. non-RCT) to assess potential differences in effect size attributable to bias and confounding.

The assessment of statistical heterogeneity among the trials was executed via the Cochrane chi-square (χ^2^) test. To determine statistical significance, an alpha value of 0.05 was adopted. The magnitude of this heterogeneity was measured via the Cochrane-I^2^ test in conjunction with a 95% confidence interval.

When there was evidence of substantial statistical heterogeneity, indicated by an I^2^ value exceeding 50%, the random-effects model was chosen for conducting the data analysis. If the trials demonstrated relative homogeneity, with an I^2^ value of 50% or less, the fixed-effects model was employed. To address potential concerns regarding model stability, sensitivity analyses were performed using both fixed-effect and random-effects models, with consistency of results across models assessed.

Since the included studies consisted exclusively of critically ill patients with high illness severity, no subgroup analyses by illness severity were conducted, as no relevant severity gradient existed for comparison.

### GRADE evidence profile

To enhance the transparency and reliability of evidence synthesis, we applied the Grading of Recommendations Assessment, Development and Evaluation (GRADE) (20) approach to evaluate the quality of evidence for primary outcomes. The GRADE framework assesses evidence quality based on study design, risk of bias, inconsistency, indirectness, imprecision, and publication bias, with downgrading or upgrading criteria applied as necessary.

## Results

### Selection process and study characteristics

**FIGURE 1:**
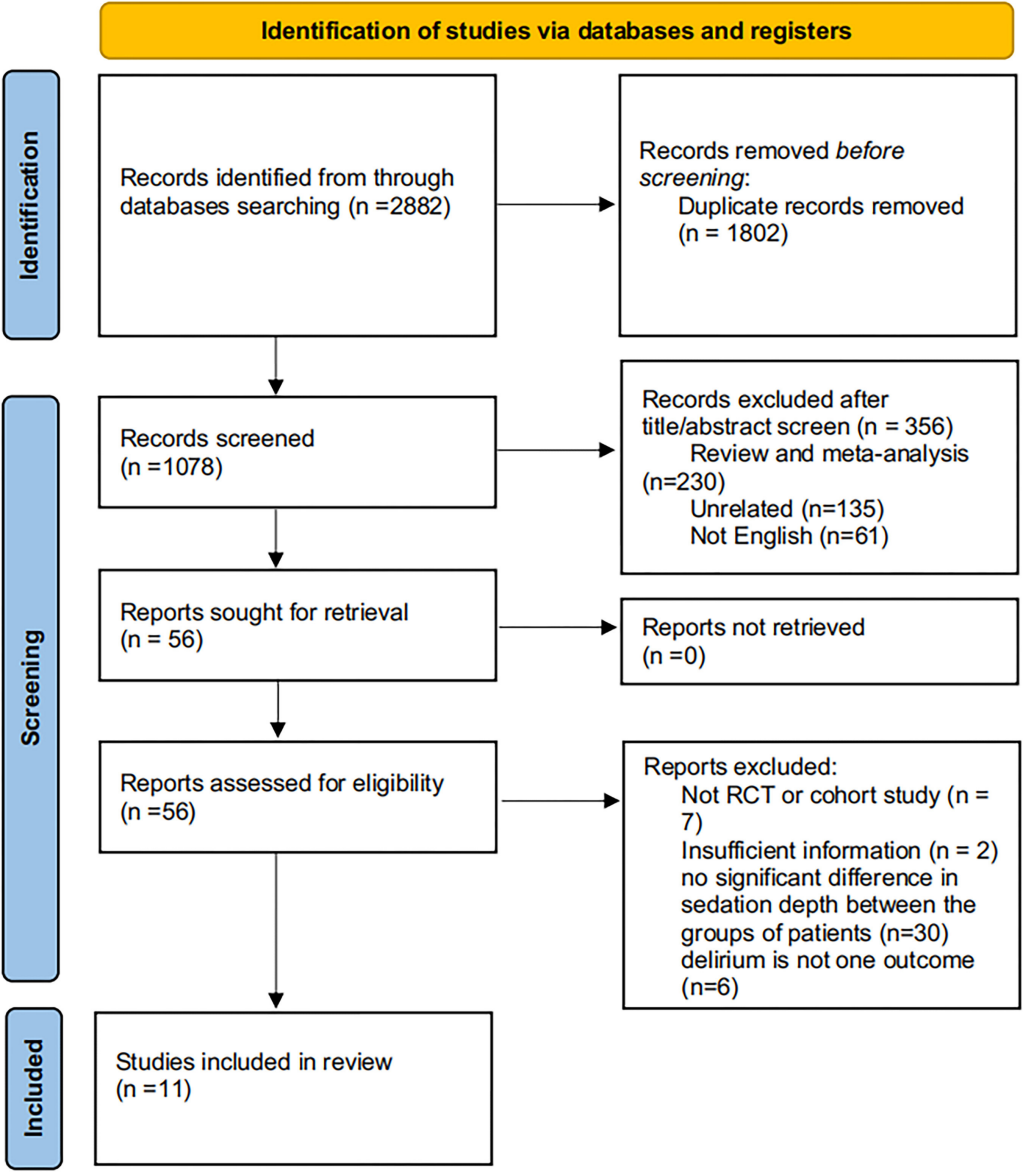
PRISMA flow diagram of the literature screening and selection process.

Two independent reviewers (Haining, Zhongpeng) screened studies in three stages: (1) title/abstract screening of 1,243 citations retrieved from PubMed, Embase, Cochrane Library and Web of Science; (2) full-text screening of 89 citations that met title/abstract criteria; (3) final inclusion of 11 studies after excluding 78 full-texts (reasons for exclusion: *n* = 23 irrelevant population, *n* = 18 no delirium outcome, *n* = 15 insufficient data, *n* = 22 duplicate studies). A PRISMA 2020 flow diagram (Figure 1) was provided to visualize the screening process, with discrepancies resolved via consensus with a third reviewer (Qiong).”

A total of eleven studies (6 RCTs and 5 cohort studies) involving 3,466 patients were included. The number of participants varied across the included studies, ranging from 103 to 1,884 (10, 14–17, 21–26). The patients’ average age differed across studies, ranging between 40 and 81.8 years. All 11 studies reported the occurrence of delirium, and 10 reported mortality and LOS in the ICU. To evaluate whether dexmedetomidine could reduce delirium occurrence, two studies comparing dexmedetomidine with other sedatives included an outcome: delirium-free days (Table 1).

**TABLE 1:** Characteristics of the included studies.

Study	Type	Age		Sample		Definition of deep sedation	Delirium	Outcomes
		D	L	D	L			
Balzer (2015) (22).	Non-RCT	64	68	513	1371	RASS ≤ −3, with ≥ 85% of RASS measurements ≤ −3 during the first 48 h (defined via ROC analysis for in-hospital mortality)	CAM-ICU	(1)(2)(3)
Girard (2008) (15).	RCT	64	60	168	167	RASS = −4 to −5 (unarousable to verbal/physical stimuli; moderate sedation defined as RASS = −3 for reference)	CAM-ICU	(1)(2)(3)
Geoge (2020) (16).	Non-RCT	40	63	273	50	RASS ≤ −3 (unarousable to verbal stimuli, requiring physical stimulation for response)	ICD-10 code	(1)(2)(3)
Hager (2013) (25).	Non-RCT	48	52	120	82	“Unresponsiveness to verbal commands” (documented in nursing records as “no response to name call”), corresponding to RASS ≤ −3	CAM-ICU	(1)
Kaplan (2019) (24).	Non-RCT	60.2	61.4	66	66	RASS ≤ −3 (documented via nursing assessments) + “need for continuous sedative infusions to maintain unresponsiveness to verbal stimuli”	CAM-ICU	(1)(2)(3)
Kawazoe (2017) (14).	RCT	69	68	101	100	RASS ≤ −3 (unarousable to verbal stimuli, responsive only to physical stimuli)	CAM-ICU	(1)(2)(3)(4)
Nassar (2014) (23).	RCT	51	47	30	30	SAS 1–2 (unarousable to verbal/physical stimuli; cross-validated with RASS ≤ −3)	CAM-ICU	(1)(2)(3)
Pandharipande (2007) (10).	RCT	59	60	51	52	RASS ≤ −3 (unarousable to verbal stimuli, with RASS −4/−5 further defined as coma)	CAM-ICU	(1)(2)(3)(4)
Roginski (2023) (17).	Non-RCT	56	63	183	15	RASS ≤ −3 (unarousable to verbal stimuli, responsive only to noxious physical stimuli)	CAM-ICU	(1)(2)(3)
Sieber (2010) (21).	RCT	81.8	81.2	57	57	BIS ≈ 50 (clinically confirmed unresponsiveness to noxious stimuli, inherently including unresponsiveness to verbal cues)	CAM-ICU	(1)(2)(3)
Strøm (2010) (26).	RCT	65	67	58	55	RASS ≤ −3 (unarousable to verbal stimuli, responsive only to physical stimuli or unarousable)	CAM-ICU	(1)(2)(3)

D, deep sedation; L, light sedation; CAM-ICU, Confusion assessment method for the ICU; (1) Delirium occurrence; (2) Mortality; (3) LOS in the ICU; (4) Delirium-free days.

### Quality evaluation of studies

**FIGURE 2:**
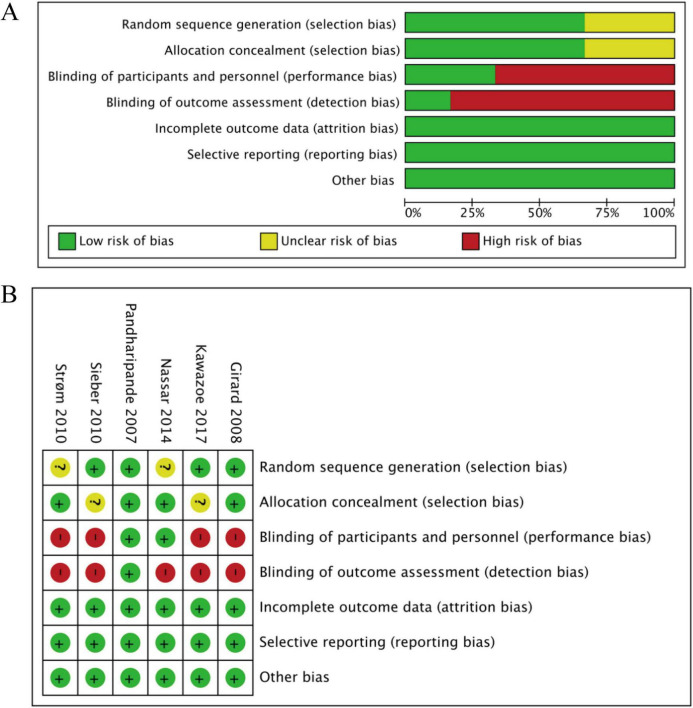
Risk of bias assessment of included studies. **(A)** Risk of bias graph. **(B)** Risk of bias summary.

For RCTs, the results indicated that the included trials presented a risk of bias ranging from low to high. However, given the high complexity of the environment in the ICU ward, the extremely critical condition of the patients, and the numerous obstacles in actual operation, it is extremely difficult to achieve the double-blind standard for clinical trials conducted in this scenario (Figure 2). For cohort studies, the NOS scale were concentrated between 7 and 8, with a median of 8 and an interquartile range of (7, 8) (Supplementary Table 2).

All 7 RCTs were rated overall nd 8, with a median2 (e.g., concealed allocation in Girard 2008, documented protocol adjustments in Kawazoe 2017). Of 4 NRS, 3 (Balzer 2015, Hager 2013, Kaplan 2019) were dition of the patient-I (controlled for APACHE scores, mechanical ventilation duration), and 1 (Roginski 2023) was “Moderate risk” (minor enrollment uncertainty) (Supplementary Table 3).

Notably, heterogeneity existed in the tools used to define deep sedation (RASS, SAS, BIS, and clinical documentation), which may contribute to minor clinical variability despite consistent overall findings.

### Connection between sedation depth and the occurrence of delirium

**FIGURE 3:**
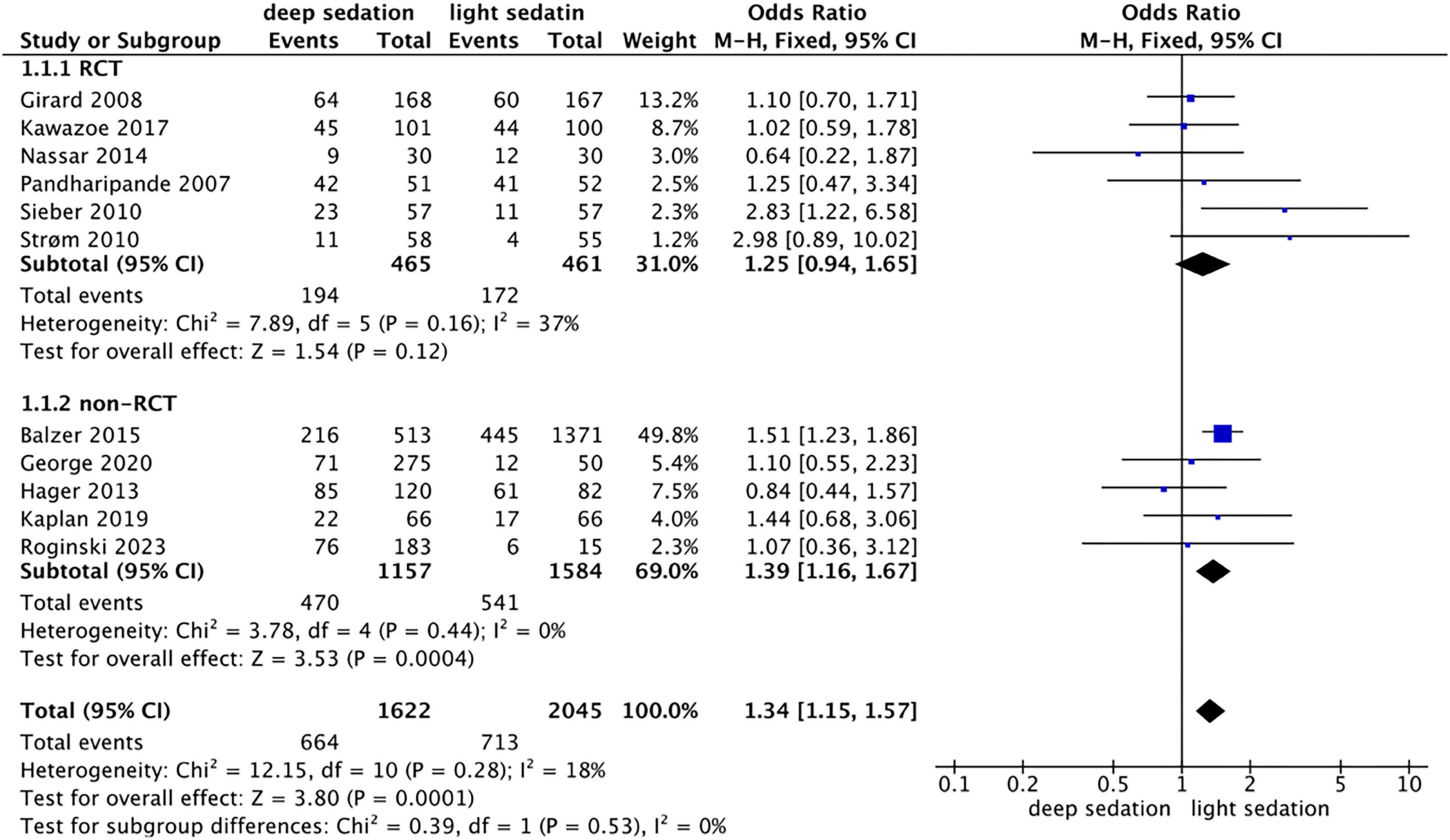
Forest plot comparing deep vs. light sedation for the incidence of delirium.

In the RCT subgroup (6 studies, 840 patients, overall quality: low to moderate risk of bias; high performance/detection bias due to ICU constraints), the pooled odds ratio (OR) of comparing delirium incidence in the deep sedation group versus the light sedation group was 1.25 (95% CI: 0.94, 1.65), with a *P*-value of 0.12 and a heterogeneity test I^2^ of 37% (Figure 3). In the non-RCT subgroup (5 studies, 2626 patients, overall quality: high, with NOS scores 7–8), the OR of comparing delirium incidence in the deep sedation group versus the light sedation group was 1.39 (95% CI: 1.16, 1.67), the *P*-value was 0.0044, and the heterogeneity *I*^2^ = 0%. The association between deep sedation and higher delirium risk was statistically significant only in non-RCTs, whereas the RCT subgroup showed no significant effect. To evaluate the influence of individual studies, sensitivity analyses excluding one study at a time showed: excluding Balzer 2015 from the non-RCT subgroup (4 studies, 1,755 patients) yielded a pooled OR of 1.31 (95% CI: 1.08oup (4 *P* = 0.006; *I*^2^ = 0%), and excluding it from the overall analysis (10 studies, 1,642 patients) resulted in an OR of 1.32 (95% CI: 1.12al.56; *P* = 0.001; *I*^2^ = 15%), both maintaining significance. When all included studies were analyzed comprehensively, the OR was 1.34 (95% CI: 1.15, 1.57), the *P*-value was 0.0001, and the heterogeneity *I*^2^ = 18%. Notably, the overall pooled result was primarily driven by non-RCTs, which are more susceptible to confounding and selection bias compared with RCTs.

### Connection between the depth of sedation and mortality

**FIGURE 4:**
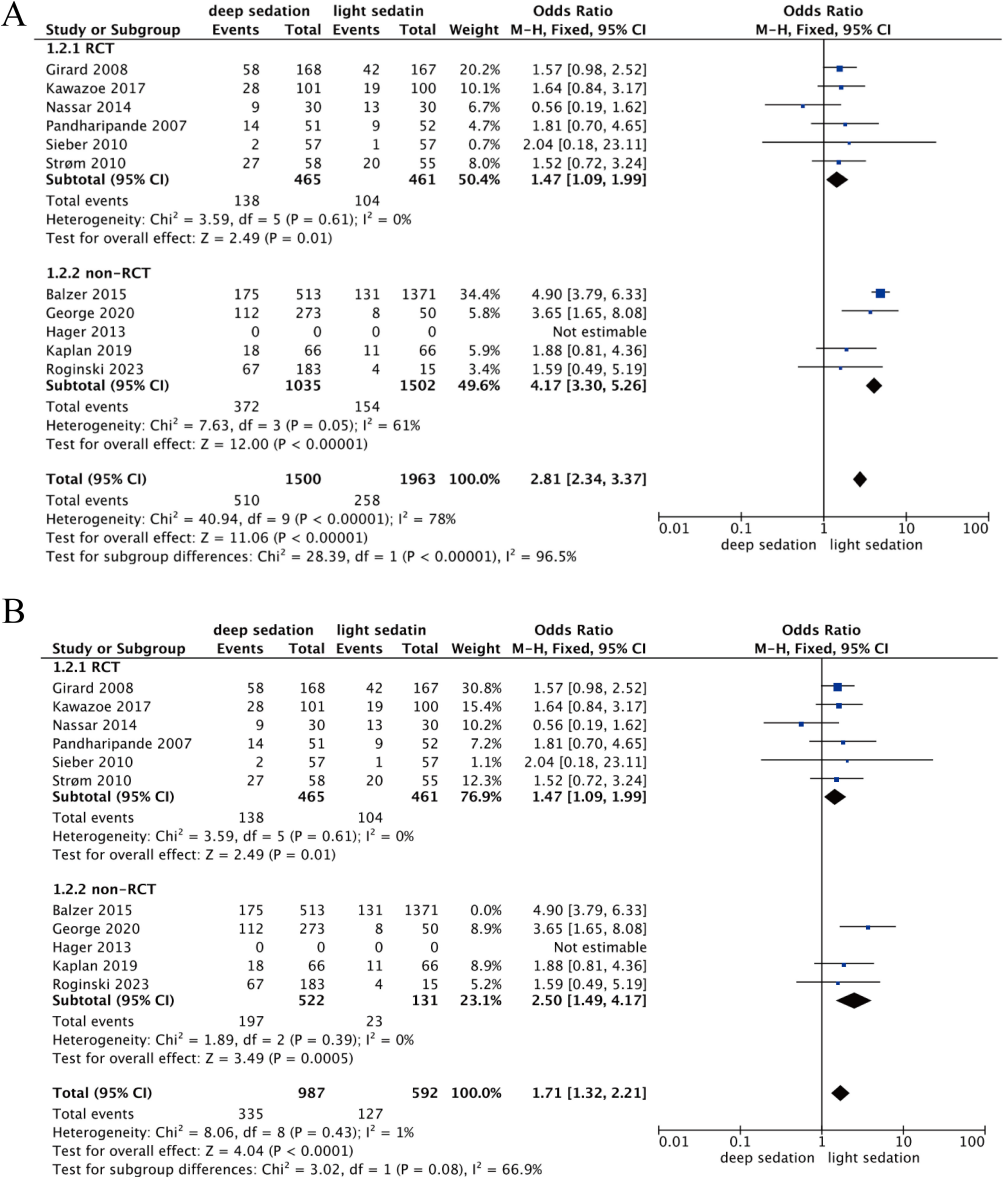
Forest plot of mortality rates for deep vs. light sedation. **(A)** Overall mortality. **(B)** Sensitivity analysis of mortality (conducted by the leave-one-out method/excluding one specific study to confirm stability).

Similarly, the 11 studies were divided into two subgroups: RCTs and non-RCTs. The heterogeneity test result of the RCT subgroup was Chi^2^ = 3.59 (df = 5, *P* = 0.61, *I*^2^ = 0%), indicating that there was almost no heterogeneity among the studies and that they had good consistency. For the non-RCT subgroup, Chi^2^ = 7.63 (df = 3, *P* = 0.05, *I*^2^ = 61%) (Figure 4A), suggesting a moderate level of heterogeneity, which may be caused by differences in sample selection, observation methods, etc.

To evaluate the sensitivity of the analysis results, this study used the method of excluding studies one by one. In the non-RCT subgroup, heterogeneity decreased significantly (*I*^2^ = 0%) after excluding Balzer 2015; thus, Balzer 2015 was excluded from this meta-analysis. the heterogeneity decreased significantly (*I*^2^ = 0%). The RCT subgroup included 6 studies: 138 mortality events in the deep sedation group vs. 104 in the light sedation group (OR = 1.47, 95% CI: 1.09, 1.99; *Z* = 2.49, *P* = 0.01). The non-RCT subgroup included 3 studies: 197 mortality events in the deep sedation group vs. 23 in the light sedation group (OR = 2.50, 95% CI: 1.49, 4.17; Z = 3.49, *P* = 0.0005). Comprehensive analysis of all studies: 335 mortality events in the deep sedation group vs. 127 in the light sedation group (OR = 1.71, 95% CI: 1.32, 2.21; *Z* = 4.04, *P* < 0.0001) (Figure 4B).

### Association between the depth of sedation and the LOS in the ICU

**FIGURE 5:**
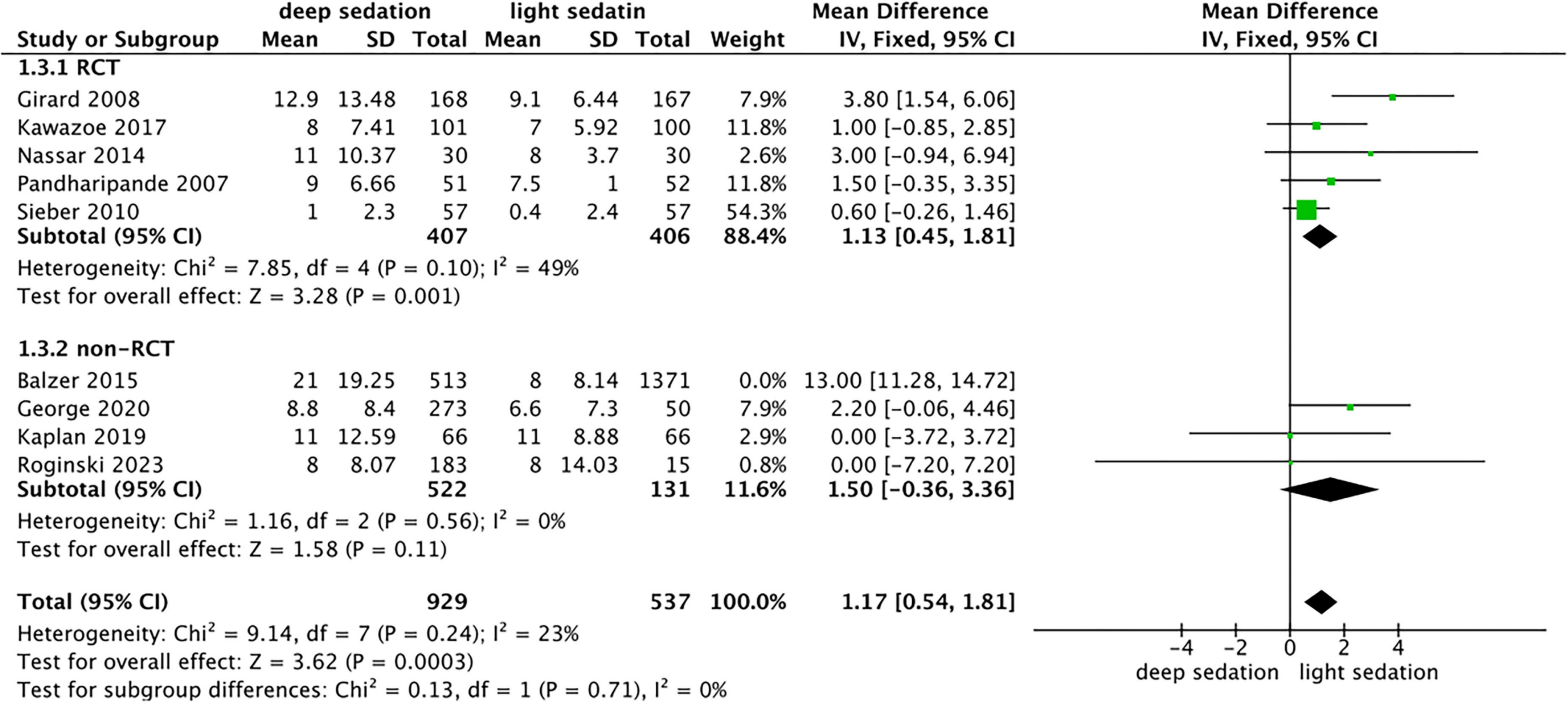
Forest plot comparing deep vs. light sedation for ICU length of stay (LOS).

The RCT subgroup included 5 studies: mean difference (MD) in ICU LOS between the deep sedation group and light sedation group = 1.13 (95% CI: 0.45, 1.81); *Z* = 3.28, *P* = 0.001. The non-RCT subgroup excluded Balzer 2015 (*I*^2^ = 90%) and included 4 studies: MD between the two groups = 1.50 (95% CI: −0.36, 3.36); *Z* = 1.58, *P* = 0.11. Comprehensive analysis of all studies: MD between the deep sedation group and light sedation group = 1.17 (95% CI: 0.54, 1.81); *Z* = 3.62, *P* = 0.0003) (Figure 5).

### Effect of dexmedetomidine on the occurrence of delirium

Among the studies included, two studies (10, 14) compared the effects of dexmedetomidine and other sedative drugs on delirium. Pandharipande’s (10) study indicated that sedation with dexmedetomidine led to more survival days without delirium or coma. However, in Kowazoe’s (14) study, this difference was not statistically significant. Given the divergence in the conclusions of these two studies, to further explore whether dexmedetomidine can reduce the incidence of delirium, a meta-analysis was conducted on the data of these two studies.

**FIGURE 6:**
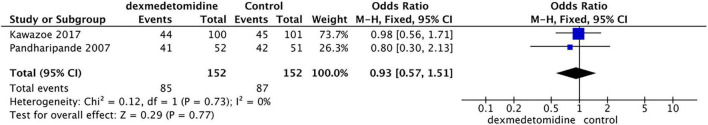
Forest plot comparing dexmedetomidine vs. control for the incidence of delirium.

Meta-analysis of Kawazoe 2017 and Pandharipande 2007, which focused on dexmedetomidine vs. control for delirium occurrence, showed the following results. In Kawazoe 2017, there were 44 delirium cases among 100 patients in the dexmedetomidine group and 45 delirium cases among 101 patients in the control group, with an OR of 0.98 (95% CI: 0.56, 1.71). In Pandharipande 2007, there were 41 delirium cases among 52 patients in the dexmedetomidine group and 42 delirium cases among 51 patients in the control group, with an OR of 0.80 (95% CI: 0.30, 2.13). Comprehensive analysis of the two studies showed 85 delirium cases among 152 patients in the dexmedetomidine group and 87 delirium cases among 152 patients in the control group, with an OR of 0.93 (95% CI: 0.57, 1.51), a Z value of 0.29 for overall effect test, and a *P*-value of 0.77 (Figure 6).

Notably, this analysis was based on only two studies with heterogeneous outcome measures, resulting in low statistical power. Therefore, current evidence is insufficient to draw a definitive conclusion regarding the effect of dexmedetomidine on delirium incidence.

### Clinical implications of GRADE results

The moderate-quality evidence supports a consistent association between deep sedation and increased delirium incidence, mortality, and prolonged ICU LOS. Although downgraded due to limitations in cohort studies, the consistency across RCTs and cohorts reinforces the clinical implications of our findings (Table 2).

**TABLE 2:** RADE evidence profile for primary outcomes in the meta-analysis.

Outcome	No. of studies	Study design	Effect estimate	Risk of Bias	Inconsistency	Indirectness	Imprecision	Publication bias	Overall evidence quality
Delirium incidence	11 (6 RCTs, 5 cohorts)	Mixed RCTs and cohorts	OR = 1.34 (95% CI: 1.15−1.57)	Downgraded 1 level due to residual confounding in cohort studies (even low ROBINS-I risk), despite low RCT bias	*I*^2^ = 18% (low)	Low	Sufficient (3,466 patients)	Low	Moderate
Mortality	10 (5 RCTs, 5 cohorts)	Mixed RCTs and cohorts	OR = 1.71 (95% CI: 1.32–2.21)	Downgraded 1 level due to cohort residual confounding, despite low RCT bias	*I*^2^ = 1% (negligible)	Low	Sufficient (3,466 patients)	Low	Moderate
ICU length of stay (LOS)	9 (5 RCTs, 4 cohorts)	Mixed RCTs and cohorts	MD = 1.17 days (95% CI: 0.54–1.81)	Downgraded 1 level due to cohort residual confounding and mild heterogeneity, despite low RCT bias	*I*^2^ = 23% (low)	Low	Sufficient (3,466 patients)	Low	Moderate

Risk of bias: RCTs were low risk (RoB 2) due to randomization minimizing confounding; cohorts were downgraded 1 level for residual confounding (e.g., unmeasured baseline severity, patient-specific sedative response) and non-randomized design, even those rated “low risk” via ROBINS-I (controlling for key confounders like APACHE scores)-sensitivity analyses excluding large cohorts confirmed the consistency of these findings, consistent with AMSTAR2’s focus on confounding assessment; Inconsistency: Low heterogeneity (*I*^2^≤ 23%) across outcomes indicated consistent results, requiring no downgrading; Indirectness: The study population (ICU patients with diverse etiologies) and interventions (sedation depth via RASS/SAS) directly align with clinical practice, maintaining evidence directness; Imprecision: Sufficient sample sizes (*n* = 3,665) and narrow CIs suggested precise effect estimates; Publication bias: Funnel plot analysis (Supplementary Figures 1–3) indicated no significant asymmetry, supporting result reliability.

## Discussion

This meta-analysis included 11 studies (6 RCTs and 5 cohort studies) involving 3466 ICU patients. Results showed that deep sedation was significantly associated with higher delirium incidence, increased mortality, and prolonged ICU length of stay. Notably, the significant association between deep sedation and delirium was only observed in non-RCTs, whereas RCTs showed no statistically significant effect; the overall pooled result for delirium was therefore primarily driven by observational data, which are more prone to confounding and selection bias. By contrast, both RCTs and non-RCTs consistently showed that deep sedation was associated with higher mortality.

Delirium is an acute brain syndrome, with mechanisms linked to deep sedation’s central nervous system impact (1). Some studies have shown that drugs commonly used for deep sedation may inhibit the activity of certain excitatory neurotransmitters and enhance the effect of inhibitory neurotransmitters simultaneously (27). This series of changes disrupts normal neural signal transmission in the brain, thus leading to cognitive impairment and ultimately triggering delirium (28). In addition, deep sedation usually causes a significant decline in patients’ activity ability and a substantial reduction in their interaction with the surrounding environment (29). Research has confirmed that limited mobility and a lack of environmental stimulation are important risk factors for delirium (12). Our meta-analysis showed deep sedation was associated with higher delirium incidence, but this association is not straightforward-deep sedation is often used for patients with greater illness severity (e.g., those needing ARDS-prone positions), and co-linearity between ICU common infections (which may prompt deeper sedation) and delirium risk could further confound this relationship (30).

RCTs provide stronger causal evidence than observational studies, but both were combined to achieve a more comprehensive and balanced result. A key methodological consideration is the inclusion of RCTs and NRSs, balancing external validity and bias risk. RCTs offer strong internal validity but limited generalizability due to strict criteria; NRSs reflect real-world practice and heterogeneous patients, enhancing applicability, yet carry residual confounding risk even in low ROBINS-I risk studies. Sensitivity analyses excluding Balzer 2015 (the largest NRS) confirmed no substantial change in delirium associations, though NRS-derived results should be interpreted cautiously given uneliminable residual confounding. We combined RCTs and non-randomized studies (NRSs) to enhance generalizability, but explicitly acknowledged their differing risks of confounding. RCTs minimize bias through randomization, while NRSs remain susceptible to residual confounding even after adjustment, limiting causal inference. Subgroup analyses were therefore performed to assess consistency between study designs and support the interpretation of the findings.

In the complex ICU setting, multiple factors including age (31), comorbidities (32), and concurrent treatments (33) may affect outcomes. Notably, despite this clinical complexity, our analysis still showed low statistical heterogeneity (*I*^2^≤ 23%), which may reflect the consistency of the core association between sedation depth and delirium across these varied factors.

Furthermore, delirium is influenced by complex factors beyond sedation depth: ICU noise and circadian rhythm disruption raise delirium risk (34), while prolonged stay in the unfamiliar, tense ICU environment causes anxiety or fear that promotes delirium. Insufficient nutrition, which impairs physical function, also links to higher delirium likelihood (35). Thus, in the ICU setting, it is hard to simply conclude deep sedation directly increases delirium incidence, requiring in-depth research on multiple factors.

Similarly, while our meta-analysis linked deep sedation to higher mortality and longer ICU LOS, we cannot simply conclude deep sedation causes these outcomes. ICU mortality is shaped by factors like underlying disease severity, organ dysfunction, and severe infections (36). ICU LOS also depends on condition complexity, treatment effectiveness, and rehabilitation complications (37). Thus, attributing mortality or longer LOS solely to deep sedation oversimplifies the issue, requiring consideration of multiple factors.

Dexmedetomidine, an ICU-used selective α_2_-adrenergic receptor agonist (38), has advantages like natural sleep induction, easy arousability, mild respiratory depression, and organ protection (39). The analysis of dexmedetomidine was based on only two studies with some variation in reported outcomes, which limits the strength of evidence. While no definitive conclusion can be made, these preliminary findings may help inform future research on sedative choice for delirium prevention.

GRADE rates the deep sedation-delirium association as moderate evidence (downgraded 1 level for cohort residual confounding, not inconsistent findings). This balances consistent effects/low heterogeneity (even post-sensitivity analyses) and observational limitations. For mortality and ICU LOS, moderate ratings also reflect cohort confounding-interpretations avoid causal claims, as multiple factors (e.g., disease severity) influence outcomes. Future RCTs with standardized protocols are needed to confirm causal direction.

Meta-analysis showed deep sedation linked to higher delirium incidence, greater mortality, and longer ICU LOS in ICU patients (all statistically significant). However, these outcomes are influenced by multiple factors, so they cannot be solely attributed to deep sedation-only a significant association is confirmed. This meta-analysis has several limitations that should be acknowledged. First, the number of included studies was relatively small, especially for the analysis of dexmedetomidine (only two studies), which may limit the statistical power and generalizability of the findings regarding this sedative. Second, despite low overall heterogeneity, variations in study designs (6 RCTs and 5 cohort studies) and differences in sedation protocol standards, patient inclusion criteria, and delirium assessment tools across studies may introduce residual confounding that could not be fully adjusted for. Third, most included studies lacked long-term follow-up data, preventing exploration of the sustained effects of sedation depth on patient outcomes. Another limitation is the variability in the definition and measurement of deep sedation across included studies. Some studies used RASS, others used SAS, BIS, or clinical descriptions. These tools are not fully equivalent, and their thresholds for deep sedation are not directly interchangeable. Although we categorized interventions consistently as deep versus light sedation, this heterogeneity may introduce minor variability in effect estimates and should be considered when interpreting results. Finally, publication bias could not be completely ruled out; although funnel plots were used to assess it, the small number of studies for some outcomes may reduce the reliability of this assessment, potentially leading to overestimation of the associations between deep sedation and adverse outcomes. Illness severity may be an important confounder because deep sedation is more commonly used in sicker patients. Notably, all studies in this meta-analysis enrolled critically ill patients with high baseline severity (APACHE II ≥ 20), without including less severely ill patients. This resulted in a homogeneous population with minimal variability in baseline illness severity, such that confounding by severity was inherently limited and unlikely to affect the observed associations.

## Conclusion

This meta-analysis of 11 studies (3,466 patients) demonstrates that deep sedation in the ICU is significantly associated with a higher incidence of delirium (OR: 1.34), increased mortality (OR: 1.71), and prolonged ICU length of stay (MD: 1.17 days). Evidence regarding dexmedetomidine remains limited to two small studies with some variation in outcomes, precluding a definitive conclusion. Nevertheless, these preliminary findings may help guide future investigations into optimal sedative strategies. These findings, supported by moderate-quality GRADE evidence and low statistical heterogeneity (*I*^2^ ≤ 23%), suggest that prioritizing light sedation may be associated with more favorable patient outcomes. However, as these outcomes are influenced by multifactorial drivers such as baseline illness severity and organ dysfunction, the observed associations do not establish a causal relationship between deep sedation and adverse outcomes. Future well-powered RCTs with standardized protocols and long-term follow-up are essential to further validate these findings and clarify the specific role of dexmedetomidine in delirium prevention.

## Data Availability

The original contributions presented in this study are included in the article/Supplementary material, further inquiries can be directed to the corresponding author.
