# Supplementary material for: The impact of sedation depth on the occurrence of delirium and prognosis in intensive care unit patients: a meta-analysis
Source: Front Med (Lausanne). 2026 May 4;13:1823354. doi: 10.3389/fmed.2026.1823354 (PMC13180737; doi:10.3389/fmed.2026.1823354)
Supplement: Supplementary file 1 [file Data_Sheet_1.docx]

Supplementary

Table S1. Search strategies

| Keywords: Delirium, Delirium of Mixed Origin, Mixed Origin Delirium, Mixed Origin Deliriums, Subacute Delirium, Deliriums, Subacute, Delirium, Subacute, Subacute Deliriums, Deep Sedation, Deep Sedations, Sedations, Deep, Sedation, Deep, Ramsay Sedation Scale, Richmond Agitation-Sedation Scale, Sedation-Agitation Scale. | | | |
| --- | --- | --- | --- |
| Pubmed | | | |
| Set | Search terms | Search type | Results |
| #1 | "Delirium"[MeSH Terms] OR "Delirium"[Title/Abstract] OR "delirium of mixed origin"[Title/Abstract] OR ((("Mixed"[All Fields] OR "mixes"[All Fields] OR "mixing"[All Fields] OR "mixings"[All Fields]) AND ("Origin"[All Fields] OR "originate"[All Fields] OR "originated"[All Fields] OR "originates"[All Fields] OR "originating"[All Fields] OR "origination"[All Fields] OR "originations"[All Fields] OR "origins"[All Fields])) AND "Delirium"[Title/Abstract]) OR ((("Mixed"[All Fields] OR "mixes"[All Fields] OR "mixing"[All Fields] OR "mixings"[All Fields]) AND ("Origin"[All Fields] OR "originate"[All Fields] OR "originated"[All Fields] OR "originates"[All Fields] OR "originating"[All Fields] OR "origination"[All Fields] OR "originations"[All Fields] OR "origins"[All Fields])) AND "Deliriums"[Title/Abstract]) OR "subacute delirium"[Title/Abstract] OR (("Delirium"[MeSH Terms] OR "Delirium"[All Fields] OR "delirium s"[All Fields] OR "Deliriums"[All Fields]) AND "Subacute"[Title/Abstract]) OR (("Delirium"[MeSH Terms] OR "Delirium"[All Fields] OR "delirium s"[All Fields] OR "Deliriums"[All Fields]) AND "Subacute"[Title/Abstract]) OR (("Subacute"[All Fields] OR "subacutely"[All Fields]) AND "Deliriums"[Title/Abstract]) | Advanced | 25943 |
| #2 | "Deep Sedation"[MeSH Terms] OR "Deep Sedation"[Title/Abstract] OR "deep sedations"[Title/Abstract] OR (("sedate"[All Fields] OR "sedated"[All Fields] OR "sedating"[All Fields] OR "Sedation"[All Fields] OR "Sedations"[All Fields]) AND "Deep"[Title/Abstract]) OR "sedation deep"[Title/Abstract] OR "ramsay sedation scale"[Title/Abstract] OR "richmond agitation sedation scale"[Title/Abstract] OR "sedation agitation scale"[Title/Abstract] | Advanced | 5332 |
| #3 | #1 AND #2 | Advanced | 622 |
| Embase | | | |
| #1 | delirium:ab,ti OR 'delirium of mixed origin':ab,ti OR 'mixed origin delirium':ab,ti OR 'mixed origin deliriums':ab,ti OR 'subacute delirium':ab,ti OR 'deliriums, subacute':ab,ti OR 'delirium, subacute':ab,ti OR 'subacute deliriums':ab,ti | Advanced | 35779 |
| #2 | 'deep sedation':ab,ti OR 'deep sedations':ab,ti OR 'sedations, deep':ab,ti OR 'sedation, deep':ab,ti OR 'ramsay sedation scale':ab,ti OR 'richmond agitation-sedation scale':ab,ti OR 'sedation-agitation scale':ab,ti | Advanced | 5667 |
| #3 | #1 AND #2 | Advanced | 780 |
| Web of Science | | | |
| #1 | (TS=(Delirium) OR AB=(Delirium OR Delirium of Mixed Origin OR Mixed Origin Delirium OR Mixed Origin Deliriums OR Subacute Delirium OR Deliriums, Subacute OR Delirium, Subacute OR Subacute Deliriums) ) | Advanced | 44655 |
| #2 | (TS=(Deep Sedation) OR AB=(Deep Sedation OR Deep Sedations OR Sedations, Deep OR Sedation, Deep OR Ramsay Sedation Scale OR Richmond Agitation-Sedation Scale OR Sedation-Agitation Scale)) | Advanced | 8001 |
| #3 | (TS=(Delirium) OR AB=(Delirium OR Delirium of Mixed Origin OR Mixed Origin Delirium OR Mixed Origin Deliriums OR Subacute Delirium OR Deliriums, Subacute OR Delirium, Subacute OR Subacute Deliriums) ) AND (TS=(Deep Sedation) OR AB=(Deep Sedation OR Deep Sedations OR Sedations, Deep OR Sedation, Deep OR Ramsay Sedation Scale OR Richmond Agitation-Sedation Scale OR Sedation-Agitation Scale)) | Advanced | 984 |
| Cochrane Library | | | |
| #1 | (Delirium OR Delirium of Mixed Origin OR Mixed Origin Delirium OR Mixed Origin Deliriums OR Subacute Delirium OR Deliriums, Subacute OR Delirium, Subacute OR Subacute Deliriums):ti,ab,kw | Advanced | 6589 |
| #2 | (Deep Sedation OR Deep Sedations OR Sedations, Deep OR Sedation, Deep OR Ramsay Sedation Scale OR Richmond Agitation-Sedation Scale OR Sedation-Agitation Scale):ti,ab,kw | Advanced | 4105 |
| #3 | #1 AND #2 | Advanced | 496 |

Table S2. NOS score

| Author, year | Representativeness of the Exposed Cohort | Selection of the Non-Exposed Cohort | Ascertainment of Exposure | Demonstration That Outcome of Interest Was Not Present at Start of Study | Comparability of Cohorts on the Basis of the Design or Analysis | Assessment of Outcome | Was Follow-Up Long Enough for Outcomes to Occur | Adequacy of Follow Up of Cohorts | Total |
| --- | --- | --- | --- | --- | --- | --- | --- | --- | --- |
| Geoge 2020 | A/1 | A/1 | A/1 | A/1 | B/1 | B/1 | A/1 | A/1 | 8 |
| Balzer 2015 | B/1 | A/1 | A/1 | A/1 | N/0 | B/1 | A/1 | B/1 | 7 |
| Roginski 2023 | B/1 | A/1 | A/1 | A/1 | B/1 | B/1 | A/1 | A/1 | 8 |
| Kaplan 2019 | B/1 | A/1 | A/1 | A/1 | N/0 | B/1 | A/1 | A/1 | 7 |
| Hager 2013 | B/1 | A/1 | A/1 | A/1 | B/1 | B/1 | A/1 | A/1 | 8 |

Table S3. Study design and Risk of Bias Assessment results of included studies

| Study | Study Design Type | Risk of Bias Assessment Tool | Risk of Bias by Domain (Domain - Risk Level) | Overall Risk of Bias | Bias Direction (RoB 2) / Target Trial Alignment (ROBINS-I) |
| --- | --- | --- | --- | --- | --- |
| Balzer 2015 | Non-Randomized Study (Matched-Pair Analysis) | ROBINS-I | Confounding bias - Low risk (matched for APACHE II score, admission type, and concurrent medications); Selection bias - Low risk; Classification of interventions - Low risk; Missing data bias - Low risk; Measurement of outcomes - Low risk; Selection of reported results - Low risk | Low risk | High alignment with target trial (intervention allocation approximated random matching; all core confounders controlled) |
| George 2020 | Non-Randomized Study (Retrospective Cohort) | ROBINS-I | Confounding bias - Low risk (adjusted for age, transport distance, and baseline neurological disease history); Selection bias - Moderate risk; Classification of interventions - Low risk; Missing data bias - Low risk (2% LOS data missing); Measurement of outcomes - Low risk; Selection of reported results - Low risk | Moderate risk | Moderate alignment with target trial (retrospective data with complete adjustment for key confounders) |
| Girard 2008 | RCT | RoB 2 | Randomization process - Low risk (allocation sequence concealed); Deviations from intended interventions - Low risk (blinded outcome assessment); Missing outcome data - Low risk (attrition rate < 5%); Measurement of outcomes - Low risk; Selection of reported results - Low risk | Low risk | Towards null (blinded design reduced outcome measurement bias; stable effect size estimation) |
| Hager 2013 | Non-Randomized Study (Pre-Post Quality Improvement) | ROBINS-I | Confounding bias - Low risk (adjusted for baseline delirium incidence and mechanical ventilation duration); Selection bias - Low risk (prospectively defined inclusion/exclusion criteria for pre- and post-intervention cohorts); Classification of interventions - Low risk; Missing data bias - Low risk; Measurement of outcomes - Low risk; Selection of reported results - Low risk | Low risk | High alignment with target trial (concurrent pre- and post-cohorts with consistent baseline characteristics; rigorous data collection) |
| Kaplan 2019 | Non-Randomized Study (Retrospective Cohort) | ROBINS-I | Confounding bias - Low risk (multivariate regression adjusted for APACHE IV score and surgical type); Selection bias - Low risk; Classification of interventions - Low risk; Missing data bias - Low risk; Measurement of outcomes - Low risk; Selection of reported results - Low risk | Low risk | High alignment with target trial (multivariate model controlled for core confounders; high data completeness) |
| Kawazoe 2017 | RCT | RoB 2 | Randomization process - Low risk; Deviations from intended interventions - Low risk (sedation protocol adjustments documented and included in sensitivity analysis); Missing outcome data - Low risk; Measurement of outcomes - Low risk; Selection of reported results - Low risk | Low risk | Towards null (documented protocol adjustments did not distort effect size; conservative bias direction) |
| Nassar 2014 | RCT | RoB 2 | Randomization process - Low risk; Deviations from intended interventions - Low risk; Missing outcome data - Low risk (10% attrition addressed via multiple imputation); Measurement of outcomes - Low risk; Selection of reported results - Low risk | Low risk | Towards null (missing data handled via valid statistical methods; minimal impact on effect size) |
| Pandharipande 2007 | RCT | RoB 2 | Randomization process - Low risk (central randomization); Deviations from intended interventions - Low risk; Missing outcome data - Low risk; Measurement of outcomes - Low risk; Selection of reported results - Low risk | Low risk | Towards null (large-sample, multicenter design; high stability of effect size estimation) |
| Roginski 2023 | Non-Randomized Study (Observational Cohort) | ROBINS-I | Confounding bias - Low risk (adjusted for gender, age, and nutritional status); Selection bias - Moderate risk; Classification of interventions - Low risk; Missing data bias - Low risk; Measurement of outcomes - Low risk; Selection of reported results - Low risk | Moderate risk | Moderate alignment with target trial (all core confounders adjusted; minor uncertainty in secondary confounders) |
| Sieber 2010 | RCT | RoB 2 | Randomization process - Low risk; Deviations from intended interventions - Low risk (double-blind design); Missing outcome data - Low risk; Measurement of outcomes - Low risk; Selection of reported results - Low risk | Low risk | Towards null (double-blind design + objective BIS monitoring; rigorous bias control) |
| Strøm 2010 | RCT | RoB 2 | Randomization process - Low risk; Deviations from intended interventions - Low risk; Missing outcome data - Low risk; Measurement of outcomes - Low risk; Selection of reported results - Low risk | Low risk | Towards null (no significant directional bias in effect size estimation) |

Figure S1. Funnel plot assessing publication bias for the association between sedation depth and delirium incidence
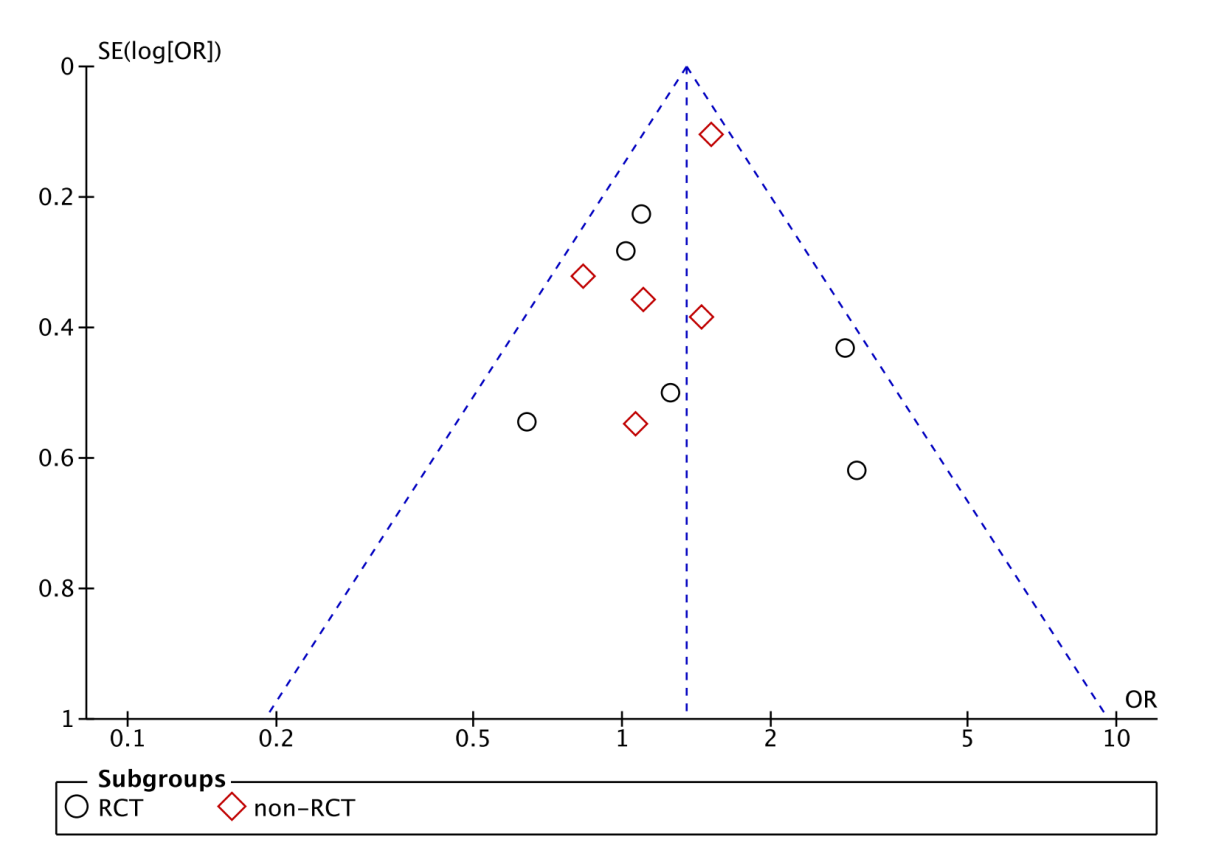


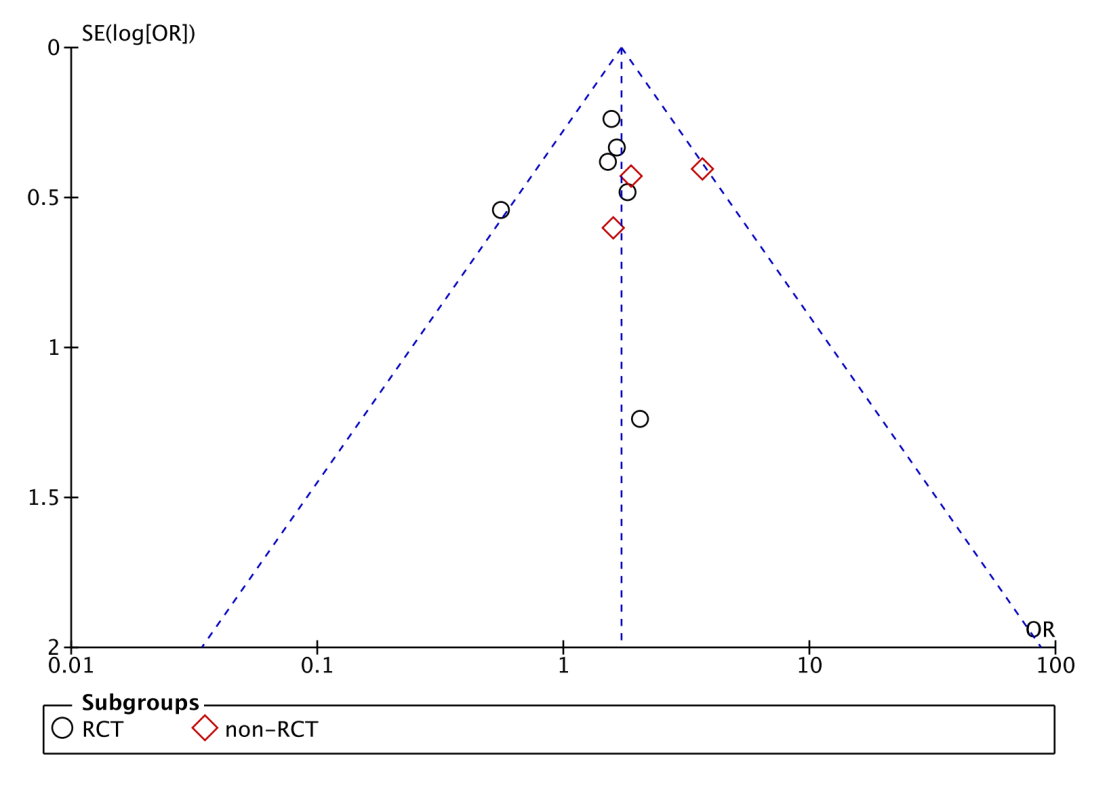


Figure S2. Funnel plot assessing publication bias for the association between sedation depth and mortality


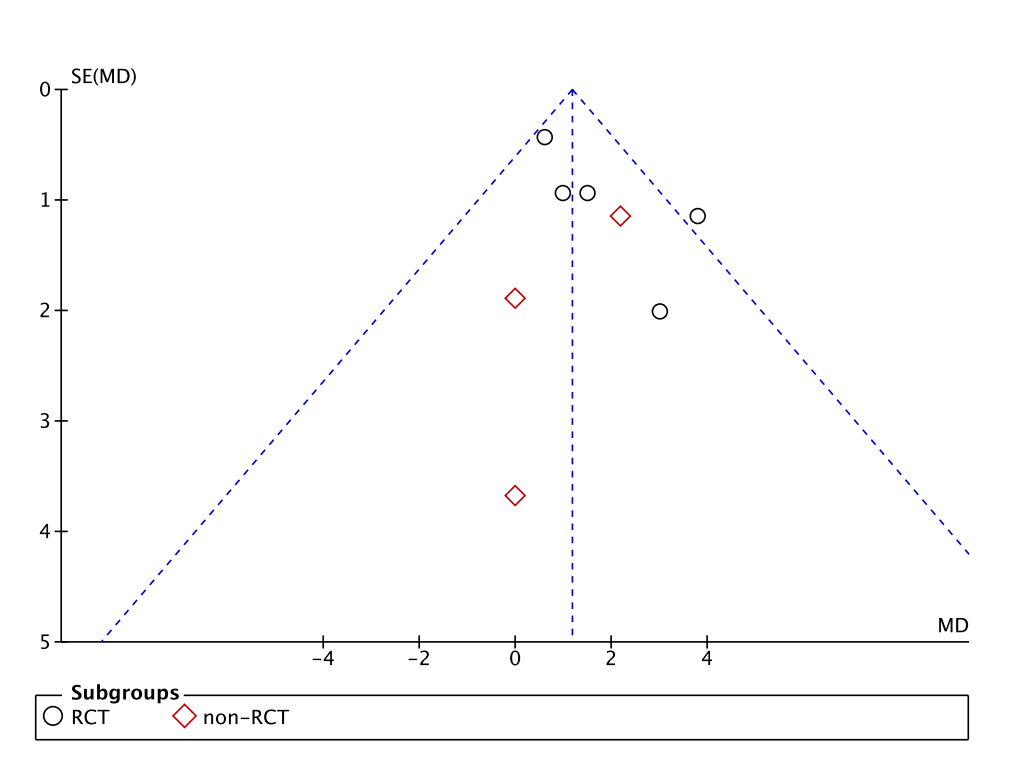


Figure S3. Funnel plot assessing publication bias for the association between sedation depth and LOS
